# Cortical Control of Striatal Dopamine Transmission via Striatal Cholinergic Interneurons

**DOI:** 10.1093/cercor/bhw252

**Published:** 2016-10-17

**Authors:** Polina Kosillo, Yan-Feng Zhang, Sarah Threlfell, Stephanie J. Cragg

**Affiliations:** 1 Department of Physiology, Anatomy and Genetics, University of Oxford, Oxford, OX1 3PT, UK; 2 Oxford Parkinson's Disease Centre, University of Oxford, Oxford, OX1 3QX, UK; 3 Current address: Department of Molecular and Cell Biology, University of California, Berkeley, CA 94720, USA

**Keywords:** AMPA receptor, corticostriatal, NMDA receptor, thalamostriatal, voltammetry

## Abstract

Corticostriatal regulation of striatal dopamine (DA) transmission has long been postulated, but ionotropic glutamate receptors have not been localized directly to DA axons. Striatal cholinergic interneurons (ChIs) are emerging as major players in striatal function, and can govern DA transmission by activating nicotinic receptors (nAChRs) on DA axons. Cortical inputs to ChIs have historically been perceived as sparse, but recent evidence indicates that they strongly activate ChIs. We explored whether activation of M1/M2 corticostriatal inputs can consequently gate DA transmission, via ChIs. We reveal that optogenetic activation of channelrhodopsin-expressing corticostriatal axons can drive striatal DA release detected with fast-scan cyclic voltammetry and requires activation of nAChRs on DA axons and AMPA receptors on ChIs that promote short-latency action potentials. By contrast, DA release driven by optogenetic activation of intralaminar thalamostriatal inputs involves additional activation of NMDA receptors on ChIs and action potential generation over longer timescales. Therefore, cortical and thalamic glutamate inputs can modulate DA transmission by regulating ChIs as gatekeepers, through ionotropic glutamate receptors. The different use of AMPA and NMDA receptors by cortical versus thalamic inputs might lead to distinct input integration strategies by ChIs and distinct modulation of the function of DA and striatum.

## Introduction

Striatal dopamine (DA) transmission plays critical roles in action selection, reward-related learning, habit formation, and disorders such as Parkinson's disease and addiction. It is becoming increasingly appreciated that DA release is governed by striatal mechanisms in addition to those that govern action potential generation in substantia nigra (SN) or ventral tegmental area (VTA) (Schmitz et al. 2003; Rice et al. 2011; Sulzer et al. 2016). The axonal propagation of action potentials could be moderated by thousands of branch points found on the axonal arbor of each DA neuron (Matsuda et al. 2009; Pissadaki and Bolam 2013), and at release sites, the dynamic probability of DA release is influenced by presynaptic mechanisms including local inputs (Cragg 2003; Rice et al. 2011; Pan and Ryan 2012; Brimblecombe et al. 2015; Sulzer et al. 2016). Inputs from striatal cholinergic interneurons (ChIs) in particular are emerging as powerful players. ACh, by acting at nicotinic receptors (nAChRs) on DA axons, can filter how different firing frequencies are relayed into the release of DA (Rice and Cragg 2004; Zhang and Sulzer 2004; Exley et al. 2008; Threlfell et al. 2010; Cohen et al. 2012; Patel et al. 2012) and furthermore, synchronous activity in a population of ChIs can trigger striatal DA release directly (Cachope et al. 2012; Threlfell et al. 2012), bypassing action potential generation in DA soma. We have identified further that glutamate inputs to ChIs from the predominantly parafascicular nucleus (Pf) of caudal intralaminar thalamus can gate striatal DA release through a mechanism that appears to require ionotropic glutamate receptors on striatal ChIs (Threlfell et al. 2012).

It has long been thought that cortex might be able to regulate striatal DA transmission, with facilitatory and inhibitory effects observed (Chéramy et al. 1986; Leviel et al. 1990; Moghaddam et al. 1990; Shimizu et al. 1990; Krebs et al. 1991; Moghaddam and Gruen 1991; Zhang and Sulzer 2003), but the facilitatory role of ionotropic glutamate receptors in particular has had little explanation because these receptors have not been localized to DA axons (Bernard and Bolam 1998; Chen et al. 1998). However, ChIs express a variety of glutamate receptors (Bernard et al. 1997; Küppenbender et al. 2000; Pisani et al. 2002; Bonsi et al. 2005; Berg et al. 2007; Nelson et al. 2014; Wieland et al. 2014). A monosynaptic connection between cortical input and ChIs has been suggested from a wealth of electrophysiological experiments showing modifications to ChI activity after cortical input stimulation in vivo and ex vivo (Wilson et al. 1990; Consolo et al. 1996a; Pisani et al. 2000; Reynolds and Wickens 2004; Ding et al. 2010; Sharott et al. 2012; Doig et al. 2014), but anatomical studies have suggested that these dendritic inputs are sparse (Meredith and Wouterlood 1990; Lapper and Bolam 1992; Thomas et al. 2000; Alcantara et al. 2001; Doig et al. 2014). Although historically perceived to be a sparse input, more recent retrograde tracer experiments suggest that cortical innervation might account for a significant share of inputs to ChIs (Guo et al. 2015). We explored whether cortical inputs could modulate DA transmission.

We show here that M1/M2 cortical inputs to striatum modulate DA release, requiring activation of ChIs via AMPA receptors, and downstream nAChRs. Furthermore, we show that Pf thalamic modulation of DA release via ChIs has greater dependence on NMDA receptors than cortical input. These findings indicate regulation of ChIs by thalamic versus cortical inputs through different integration strategies that might differently govern how ChIs modulate striatal function, including DA transmission.

## Materials and Methods

### Animals and Surgery

Heterozygous and homozygous adult (21–40 days) male and female CaMKIIa-Cre mice (B6.Cg-Tg(CaMK2a-Cre)T29-1Stl/J, stock 005359) were anaesthetized with isoflurane (2% w/o), and injected bilaterally with 400 nL of an adeno-associated virus backbone serotype 5 (AAV5, 8×10e^12^ particles/mL), carrying ChR2-eYFP (pAAV-EF1α-DI0-hChR2(H134R)EYFP-WPRE-pA) (Gene Therapy Center Virus Vector Core, University of North Carolina) (Boyden et al. 2005; Zhang et al. 2006; Tsai et al. 2009). Serotype AAV5 was used for good transduction efficiency and spread (Burger et al. 2004; Paterna et al. 2004; Markakis et al. 2010), and was used previously to successfully drive thalamostriatal inputs to ChIs and DA release (Threlfell et al. 2012). Injections for motor cortex (mCtx) were targeted to ML 1.7 mm, AP +1.3 mm, DV 1.0 mm from bregma. Injections targeted to the Pf nucleus of the caudal intralaminar thalamus were ML 0.7 mm, AP −2.3 mm, DV 3.5 mm from bregma. Coordinates were taken from the adult mouse brain atlas (Paxinos and Franklin 2008). Injection sites were confirmed with post hoc immunocytochemistry for eYFP expression and co-injected red polystyrene fluorescent spheres (Invitrogen). In all, 4–10 weeks were allowed for transgene expression.

### Fast-Scan Cyclic Voltammetry

DA release was monitored using fast-scan cyclic voltammetry (FCV) in acute coronal slices as used previously (Rice and Cragg 2004; Exley et al. 2008; Threlfell et al. 2010, 2012). In brief, after brain removal following cervical dislocation and decapitation, 300 μm coronal slices were cut on a vibratome (Leica VT1200S) in ice-cold HEPES-buffered artificial cerebrospinal fluid (aCSF) saturated with 95% O_2_/5% CO_2_. Slices between +1.5 mm and +0.5 mm from bregma containing caudate-putamen and nucleus accumbens (i.e. striatum) were used for experimentation (Paxinos and Franklin 2008). In the recording chamber, slices were maintained at 32 °C in aCSF containing in mM: 130 NaCl, 25 NaHCO_3_, 2.5 KCl, 1.25 NaH_2_PO_4_, 2 CaCl_2_, 2 MgCl_2_, 10 glucose. Striatal DA release following light activation of ChR2-expressing fibers was monitored with FCV at carbon-fiber microelectrodes (CFMs) using a Millar voltammeter (Julian Millar, Barts and the London School of Medicine and Dentistry). CFMs were fabricated in-house from epoxy-free carbon fiber 7–8 μm in diameter (Goodfellow Cambridge Ltd) enclosed in glass capillary and cut to final tip length of 50–100 μm. A triangular waveform was applied to carbon fiber scanning from −0.7 V to +1.3 V and back, against Ag/AgCl reference electrode at a rate of 800 V/s. Evoked DA transients were sampled at 8 Hz, and data were acquired at 50 kHz using AxoScope 10.2 (Molecular Devices) or Whole Cell Program (WCP, University of Strathclyde, Glasgow, UK). Recorded FCV signals were identified as DA by comparing oxidation (+0.6 V) and reduction (−0.2 V) potential peaks from experimental voltammograms with currents recorded during calibration with 2 μM DA dissolved in experimental media.

### Whole-Cell Patch-Clamp Electrophysiology

Methods used for whole-cell patch clamp in acute coronal slices were as described previously (Threlfell et al. 2012). Briefly, mice were anaesthetized with pentobarbital and transcardially perfused with ice-cold high Mg^2+^ aCSF containing in mM: 85 NaCl, 25 NaHCO_3_, 2.5 KCl, 1.25 NaH_2_PO_4_, 0.5 CaCl_2_, 7 MgCl_2_, 10 glucose, 65 sucrose. Coronal 300 μm slices were cut on a vibratome (Leica VT1200S) at coordinates between +1.5 mm and +0.5 mm from bregma (Paxinos and Franklin 2008). Slices recovered at 32°C for 30–40 min after dissection and were subsequently kept at room temperature. Slices were maintained and recorded from in aCSF containing in mM: 130 NaCl, 25 NaHCO_3_, 2.5 KCl, 1.25 NaH_2_PO_4_, 2 CaCl_2_, 2 MgCl_2_, 10 glucose. The aCSF was saturated with 95% O_2_/5% CO_2_; recordings were made at 32°C.

Glass electrodes for whole-cell patch clamp (5–9 MΩ) were filled with an intracellular solution containing in mM: 120 K-gluconate, 10 KCl, 10 HEPES, 4 MgATP, 0.3 NaGTP, 10 Na-phosphocreatine and 0.5% neurobiotin. ChIs in the striatum were identified by their distinctive morphological features, that is, large somas (>20 µm) and their characteristic electrophysiological properties, that is prominent Ih, after-hyperpolarization (AHP) and broad action potential. A minimum negative holding current (<40 pA) was applied to prevent spike activity during recordings of laser-induced excitatory post-synaptic potentials (EPSPs). Recordings were acquired using a Multiclamp 700B at 10–20 kHz. All data were analyzed offline with Clampfit (pClamp10), and custom-written Matlab (R2013b) scripts. ChIs were confirmed by post hoc labelling for choline acetyltransferase (ChAT) and neurobiotin, as previously (Threlfell et al. 2012).

### Optogenetic Stimulation

ChR2-eYFP-expressing neuropil in striatum was activated using blue light pulses (473 nm, OptoLED, CAIRN or laser DL473, Rapp OptoElectronic) transmitted through the microscope objective via a fluorescence arm, pulse width 2 ms, generated out of phase with voltammetric scans. Full-field illumination with the LED system for FCV experiments covered an area 2.2 mm in diameter under ×10 immersion objective, and light power density was 25–30 mW/mm^2^. Spot laser illumination for patch-clamp experiments had a 30 µm diameter under ×40 immersion objective, and light power density between 150 µW/mm^2^ and 23 mW/mm^2^. Light power used reflected variation in ChR2-eYFP levels and the experimental measure, with lowest levels being used to probe EPSPs without evoking action potentials, and higher intensities were required to drive inputs to make cells spike. Stimulation frequencies used were 10 and 25 Hz (400 ms trains) in order to reflect the frequencies observed in vivo in input neurons during activated/arousal states for Pf and cortical inputs, respectively (Agmon and Connors 1989; Helmchen et al. 1999; Lacey et al. 2007). Since there was no effect of pulse number or frequency on DA evoked or effects of pharmacological ligands, 10 and 25 Hz data were pooled unless otherwise specified. Protocols were repeated at intervals of 2.5 min. Recordings for Pf and mCtx groups were acquired from across the CPu in an unbiased manner in relation to site location, but there was a tendency for more successful recordings in more lateral sites for mCtx injections and in more medial sites for Pf. However, the distributions of striatal recording locations for experiments that identified differences in receptor pharmacology were comparable and spanned the dorsal striatum.

### Immunohistochemistry to Verify Targeted Injections

To verify cortical injection sites, striatal coronal sections used for recordings were fixed in 4% PFA and 0.2% saturated picric acid (pH 7.2–7.4) for at least 24 h at 4°C. Sections were washed in PBS, and mounted on gelled slides using Vectashield (Vector Laboratories) for visualization. To verify thalamic injection sites, brain blocks containing thalamus were taken after preparing striatal slices used in recordings. Blocks were fixed in 4% PFA and 0.2% saturated picric acid (pH 7.2–7.4) for at least 72 h at 4°C. Blocks were washed in PBS, re-sectioned at 50–100 μm on a vibrating microtome (VT1000S, Leica Microsystems), washed in PBS (5 × 5 min) and mounted with Vectashield (Vector Laboratories). Injection sites (red spheres) and eYFP-tagged ChR2-transduced neuropil were visualized using a fluoescence microscope Olympus BX41 (Olympus medical) coupled to Q-Click cooled monochrome CCD camera and Q-capturePro 7.0 software. Red spheres confirmed targeted injections. ChR2-eYFP expression targeted to Pf thalamus was expressed most densely and predominantly in Pf with some limited expression in wider intralaminar thalamus. For simplicity, and as the center of our targeted ChR2 expression, we use the term Pf here. ChR2 expression in off-target regions could also occur including in non-injected thalamus and cortex from their reciprocal connectivity; however, we could not resolve any cell body labelling in off-target sites, and moreover, the physiological and pharmacological differences we reveal here confirm that distinct inputs to striatum are differentially activated when we target ChR2 injections to either thalamus or cortex.

To verify that recorded neurons were ChIs, neurons filled with neurobiotin were co-labelled for ChAT. Acute striatal slices were fixed after recording, in 4% paraformaldehyde dissolved in PBS containing 0.2% picric acid. Slices were fixed overnight at 4°C and then stored in PBS. Free-floating sections were then washed in PBS 5 × 5 min and incubated in 0.5% Triton X-100 and 10% normal donkey serum. Slices were subsequently incubated with goat anti-ChAT 1:100 (Millipore) antibody dissolved in PBS containing 0.5% Triton X-100 and 3% normal donkey serum overnight. Sections were then washed with PBS 5 × 5 min and incubated for 2 h at room temperature with 1:1000 Alexa Fluor 568 donkey anti-goat (Invitrogen) antibody dissolved in PBS containing 0.5% Triton X-100 and 3% normal donkey. Alexa 488-conjugated streptavidin (Invitrogen) was included in the secondary antibody solution at a final concentration of 1:250 to identify the recorded neurons. Sections then washed with PBS and mounted on gelled slides with Vectashield mounting medium (Vector Labs) and imaged using an AxioSkop fluorescent microscope (Zeiss).

### Experimental Design and Statistical Analyses

**Figure 1.**
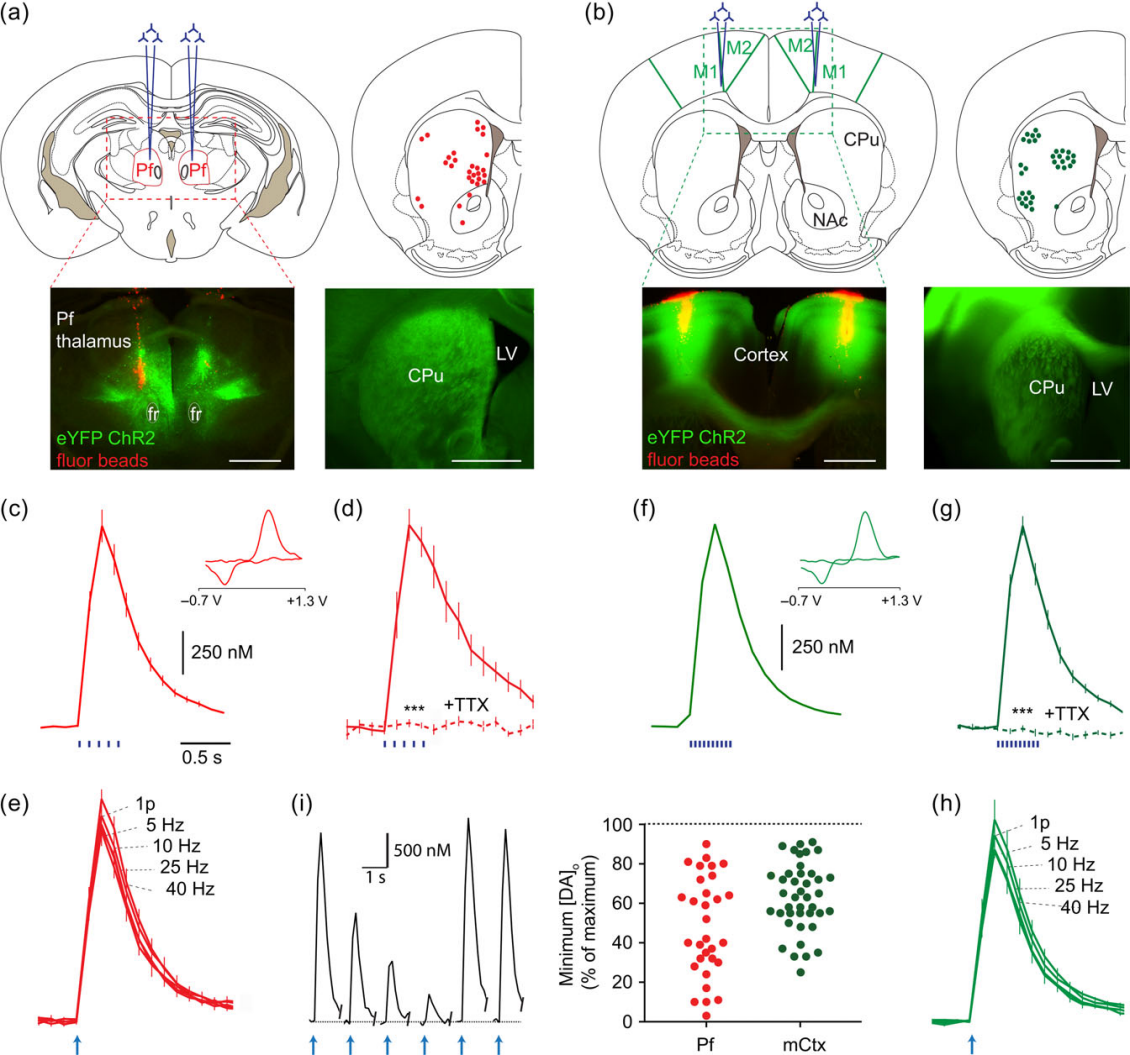
Activation of thalamic or cortical afferents to striatum drives DA release. (*a,b*) *Upper left,* Typical bilateral injection sites of AAV-packaged ChR2 and *lower left,* red fluorescent marker beads and eYFP-tagged ChR2 targetted to (*a*) Pf intralaminar thalamus or (*b*) M1/M2 cortex (*mCtx*). *Upper right*, the distribution of DA release sites sampled throughout experiments. *Lower right*, example striatal ChR2-eYFP expression. Scale, 1 mm. (*c–h*) Mean striatal [DA]_o_ ± SEM versus time after blue light stimulation of striatum from Pf- (*c*–*e*) or mCtx-injected mice (*f*–*h*). *Insets*, typical cyclic voltammograms show characteristic DA waveform. Stimuli (*blue bars*) and *n* values: (*c*) 400 ms train at 10 Hz, 23 recording sites in 11 mice; (*d*) 400 ms train at 10 Hz, 3 recording sites in 3 mice, TTX (1 µM, *dashed line*), data normalized to control; (*e*,*h*) 1 or 5p at 5–40 Hz (*arrow indicates stimulation start*), *n* = 3 mice, data normalized to 1p; (*f*) 400 ms train at 25 Hz, 30 recording sites in 17 mice; (*g*) 400 ms train at 25 Hz, *n* = 3 mice, TTX (1 µM, *dashed line*), data normalized to control. ***P < 0.001, Wilcoxon test. (*i*) Trial-by-trial reversible variability in evoked [DA]_o_ at individual recording sites. *Left,* Example excerpt of recording at one recording site, 5p/10 Hz stimuli, Pf injected, *scale bars* are for the plots, plotted at 2.5 min intervals. *Right*, Scatter plot of reversible variation shows the lowest value detected expressed as a % of maximum value seen at that site (includes sites not used for further analysis).

Data are expressed as mean ± SEM. The *n* value is the number of experiments unless otherwise stated, each conducted in a minimum of three different animals. For DA release data, we included a minimum of four release events for each stimulus or condition at individual recording sites, unless otherwise stated. Drug data were normalized to control data, and frequency data were normalized to single pulses, before collating across experiments. DA peak concentrations were statistically compared using Wilcoxon or Kruskall–Wallis non-parametric tests. For Figure 1*i*, data for release variability indicate the minimum value in release seen as a % of the maximum release seen at each release site. For EPSP data, the maximum EPSP amplitude and the area under the curve were normalized to the control condition within each group. Data were compared with the control condition (hypothetical value 1.000, i.e. the normalized control group) using a one-sample *t*-test.

### Drugs

D-APV, GYKI 52466 hydrochloride (GYKI), oxotremorine-M (Oxo-M), MCPG, bicuculline and saclofen were obtained from Tocris Bioscience or Ascent Scientific. Dihydro-β-erythroidine (DHβE) and other chemicals were obtained from Sigma Aldrich. Stock aliquots of drugs were prepared at 1000–10 000× final concentrations in de-ionized water or aqueous acid (GYKI) and stored at −20°C. None of the drugs applied altered electrode sensitivity to DA. Data from immediately prior to drug application were compared with data acquired when drug effects equilibrated, which was typically 10–20 min after application.

## Results

### Light-Activation of ChR2-Expressing Thalamic or Cortical Afferents Triggers Striatal Dopamine Release

CaMKIIa-Cre mice received injections of an AAV-packaged floxed construct for ChR2-eYFP into either Pf thalamic nucleus as previously (Threlfell et al. 2012) (Fig. 1*a*) or mCtx (Fig. 1*b*). Subsequently, ChR2-eYFP expression was detected in the striatal neuropil (Fig. 1*a,b*). In acute coronal slices containing ChR2-eYFP expression, we recorded extracellular DA concentration ([DA]_o_) using FCV at CFMs (Threlfell et al. 2012) at a range of sites from across the dorsal striatum (Fig. 1*a,b*) whilst stimulating striatum with blue light pulses. To firstly corroborate previous observations, we confirmed that activation of thalamostriatal ChR2-eYFP afferents in Pf-injected mice evoked the release of striatal DA (Fig. 1*c–e*). We then explored the effect of light-activation of ChR2-eYFP afferents in mCtx-injected mice. Light activation of corticostriatal afferents powerfully drove DA release (Fig. 1*f–h*). Light-evoked [DA]_o_ for both inputs was TTX-sensitive (Fig. 1*d,g*), Ca^2+^-dependent and approximated the levels evoked by local electrical stimulation (data not illustrated). Evoked [DA]_o_ did not vary with frequency (5–40 Hz for 5 pulses) for Pf or mCtx inputs (Fig. 1*e,h*), a feature seen following activation of ChR2-expressing ChIs (Threlfell et al. 2012). We noted however that in contrast to the activation of DA release by direct light activation of ChR2-expressing ChIs reported previously (Threlfell et al. 2012), levels of DA release evoked by thalamic or cortical inputs were not easily predicted by eYFP expression at recording sites: firstly, ChR2-eYFP expression was not always sufficient for DA release; and secondly, DA release seen after light-activation could be highly variable on a stimulus-by-stimulus basis at a given release site (Fig. 1*i*), with the minimum evoked [DA]_o_ release seen at each site varying reversibly 50–60% of maximum level seen (averaged across sites), and to as little as 5%. These observations suggest an additional process, such as an intermediary circuit between glutamate inputs and DA axons, bearing some autonomous dynamic properties that lead to its varying recruitment.

### Glutamate Inputs Drive Striatal Dopamine Release via AMPA-, NMDA-, and Nicotinic Receptors

**Figure 2.**
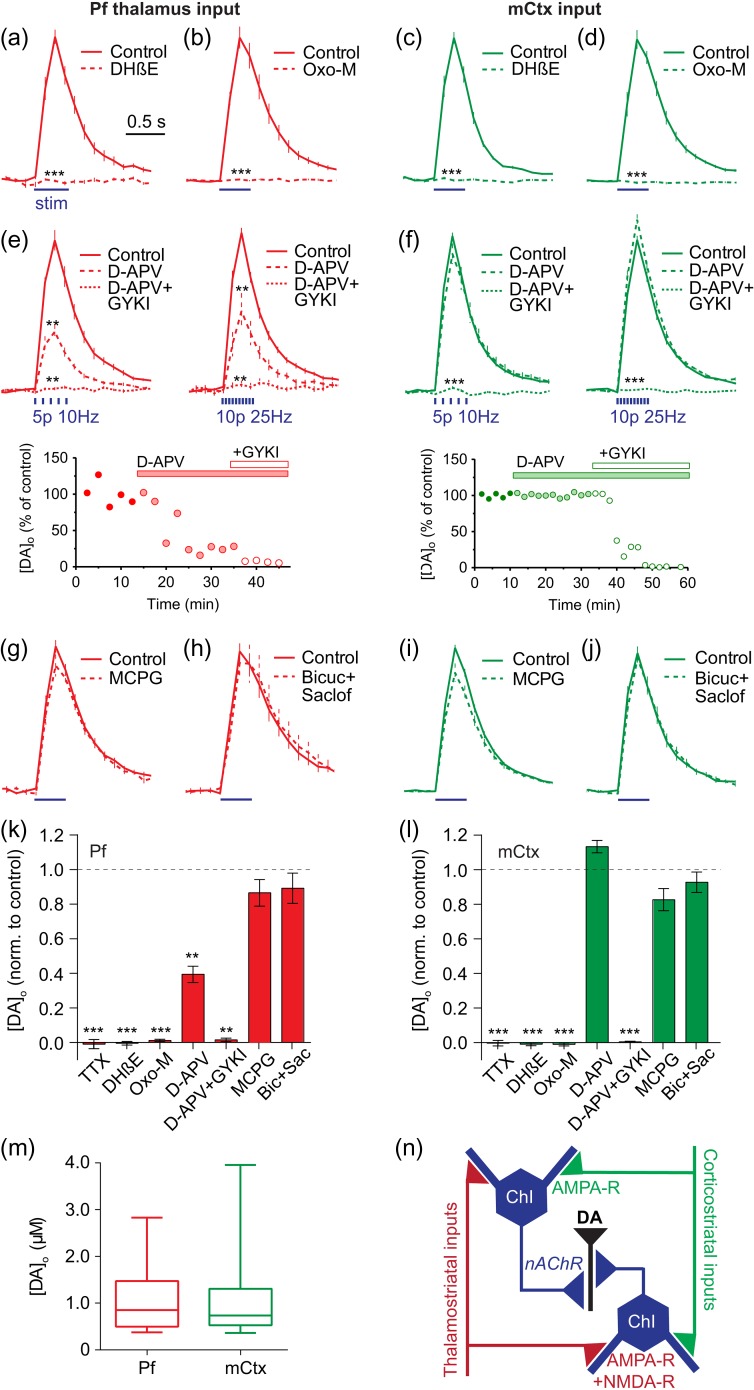
Thalamic and cortical activation of DA release requires nAChRs and distinct glutamate receptors. (**a–j**) Mean light-evoked striatal [DA]_o_ ± SEM versus time in Pf- (*a*,*b*,*e*,*g*,*h*) or mCtx-injected (*c*,*d*,*g*,*f*,*i*,*j*) mice in control conditions (*solid*) versus the presence (*dotted lines*) of: (*a,c*) DHβE (1 µM) (*n* = 3–5 mice), (*b,d*) Oxo-M (10 µM) (*n* = 3–4), (*e,f*) D-APV (50 µM) then plus GYKI (10 µM) (*n* = 4–5), (*g,i*) MCPG (200 µM) (*n* = 3–5), or (*h,j*) bicuculline (10 µM) and saclofen (50 µM) (*n* = 3–5). Data are normalized on vertical axes relative to control values prior to drug. Stimuli (*blue lines*) are 400 ms trains with 10 Hz/25 Hz pooled unless otherwise stated. (*e,f*) *Lower*, example plots of peak [DA]_o_ detected over time at individual recording sites upon application of ionotropic glutamate receptor blockers. (*k,l*) Summary of peak striatal [DA]_o_ ± SEM following drug application normalized to control. Wilcoxon tests: **P < 0.01, ***P < 0.001. (*m*) Mean, SD and range of evoked [DA]_o_ at release sites (in µM) (*n* = 23–30 recording sites in 11–17 mice). (*n*) Schematic of circuitry.

We identified the receptors involved for both thalamostriatal- and corticostriatal-evoked DA release. For both inputs, DA release required activation of nAChRs: DA release was reversibly abolished by an antagonist for β2-subunit-containing nAChRs, DHβE (1 µM) (Fig. 2*a,c*). These data indicate that ACh action at nAChRs is required for DA release driven by activation of either Pf or mCtx, implicating activation of ChIs as the common driving mechanism. DA release was also prevented by a broad-spectrum muscarinic agonist, oxotremorine-M (10 µM) (Fig. 2*b,d*), consistent with previous observations that M2 and M4-mAChRs can inhibit DA release by inhibiting ACh release from ChIs (Threlfell et al. 2010; Shin et al. 2015) and ChI activity (Ding et al. 2006), but also consistent with presynaptic inhibition of glutamate input from corticostriatal terminals by M2/M4-mAChRs (Calabresi et al. 1998; Barral et al. 1999; Alcantara et al. 2001). Furthermore, application of an NMDA receptor antagonist (D-APV, 50 µM) followed by an AMPA-receptor antagonist (GYKI, 10 µM) showed that DA release driven by Pf involved both NMDA and AMPA receptors (10 or 25 Hz, Fig. 2*e*), whereas mCtx inputs predominantly relied on AMPA receptors (10 or 25 Hz, Fig. 2*f*). Note that DA release driven in CPu by light-activation of ChIs directly does not involve glutamate receptors (Threlfell et al. 2012); and therefore, the glutamate dependence of DA release driven by Pf or mCtx does not result from glutamate potentially co-released by ChIs (Higley et al. 2011). There was no detectable effect of antagonists for metabotropic glutamate receptors (mGluRs), or GABA_A_ or GABA_B_ receptors (Fig. 2*g–l*). Mean evoked [DA]_o_ was similar following stimulation of Pf versus mCtx afferents (Fig. 2*m*).

These data suggest a disynaptic circuit in which corticostriatal and thalamostriatal glutamate inputs activate AMPA receptors on ChIs, with or without significant involvement of NMDA receptors, which, in turn, activate nAChRs on DA axons to trigger DA release (Fig. 2*n*). The variability in glutamate-evoked [DA]_o_ seen over time could be consistent with compounded thresholds of transmission from multiple neurons in a serial circuit (glutamate-ACh-DA) and also with the autonomous dynamic activity expected of the intermediary ChIs (Wilson et al. 1990; Kawaguchi 1993). Glutamate inputs might be expected to modulate only the timing of autonomous pacemaking by ChIs (Bennett and Wilson 1998), and increase but not guarantee spike probability, with a potentially variable outcome on DA.

### Glutamate Inputs Activate ChIs via Different Glutamate Receptors

**Figure 3.**
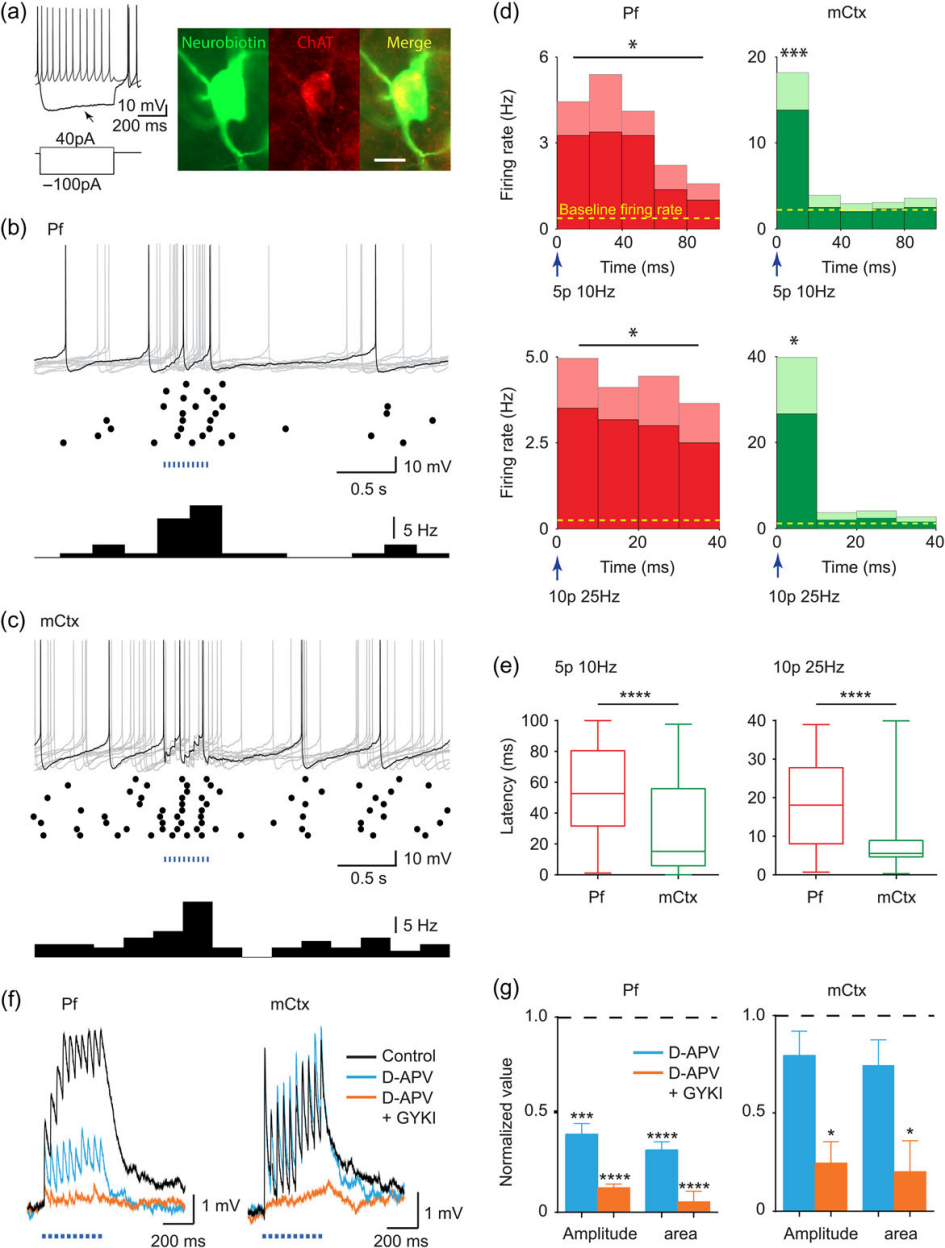
Cortical and thalamic striatal afferents drive ChI activity via ionotropic glutamate receptors. (*a*) Recorded cells show typical electrophysiological signature of ChIs with membrane potential sag (*arrow*) and co-label for neurobiotin fill (*green*) and ChAT (*red*). Anatomy scale bar, 20 µm. (*b,c*) Activity in ChIs following blue light-activation (400 ms train, 25 Hz) of (*b*) Pf or (*c*) mCtx afferents: *top,* example current clamp traces, 10 sweeps superimposed (*gray*), with 1 typical highlighted (*black*), *middle,* raster plot, 10 sweeps, *bottom*, histogram (bin size 0.25 s). (*d*) Mean spike frequency and SEM (mean, *dark shading*, and SEM, *light shading*) averaged following each light pulse in a train is significantly increased compared with mean baseline (*dotted line,* mean firing rate in the 3 s before stimulation), Pf (*left*, *n* = 4–6), mCtx (*right*, *n* = 6), 5 pulses 10 Hz (*upper*), 400 ms train at 25 Hz (*lower*). Mean baseline firing rates range from 0.252 to 2.21 Hz. Mean evoked activity corresponds to mean spike probabilities per stimulus ranging from 0.19 to 0.62. (*e*) Mean, 25% and 75% percentiles, and range of latencies to spike for 10 Hz (*left*) or 25 Hz (*right*) stimulation (400 ms train) of Pf (*left*) or mCtx (*right*) afferents. Latencies are significantly shorter for mCtx activation (Mann–Whitney test). (*f*) Example traces and (*g*) mean amplitude and area under the curve, of light-evoked EPSPs in ChIs in control conditions (*black*), in D-APV (50 µM, *blue*), then plus GYKI (10 µM, *orange*) after activation of Pf (*left*, *n* = 4–5) or mCtx afferents (*right*, *n* = 3–5). One-sample *t*-test versus control: *P < 0.05, **P < 0.01, ***P < 0.001, ****P < 0.0001.

We identified whether ChR2-expressing inputs from mCtx and Pf were activating ChIs with these protocols. Light-activation of either Pf or mCtx afferents (10 and 25 Hz) significantly increased spike probability and firing rate in individually patched ChIs (Fig. 3*a–d*) although spike probability per stimulus was <1 (unlike direct activation of ChIs; Threlfell et al 2012). Spike latency differed between inputs: mean latency to spike (averaged following all individual pulses) was short after mCtx activation (typically <10 msec) but significantly longer for Pf afferents (spanning ~60 msec) (Fig. 3*b–e*). Correspondingly, the glutamate receptor types co-varied with input type and with the receptors that regulated DA release: EPSPs generated by light-activated Pf inputs were NMDA- and AMPA-receptor mediated, whereas those activated by mCtx inputs were predominantly AMPA-receptor-mediated (Fig. 3*f,g*). These data reveal that different subsets of ionotropic glutamate receptors are recruited by Pf thalamic versus M1/M2 cortical inputs to ChIs, consistent with the receptor dependence of striatal DA release. They suggest furthermore that the time window during which ChI firing could be modified, and even synchronized, by its inputs to modulate DA release or other aspects of striatal function differs between cortical and thalamic inputs due to different postsynaptic receptors.

## Discussion

We show here that cortical glutamate input to the dorsal striatum can regulate striatal DA release via activation of ionotropic glutamate receptors on ChIs which serve as gatekeepers. The cortical input to the striatum has been viewed as providing a relatively sparse innervation of ChIs compared with thalamic Pf inputs, but we identify here that both types of input can effectively recruit ChIs, and drive DA transmission. Therefore, cortical and thalamic inputs might modify striatal function through mechanisms that extend to the regulation of striatal DA release.

The finding that glutamate inputs to striatum can modulate DA transmission via modulation of ChI activity finally provides a mechanism for earlier reports of DA efflux following glutamate application despite an absence of ionotropic glutamate receptors on DA axons (Chéramy et al. 1986; Leviel et al. 1990; Shimizu et al. 1990; Krebs et al. 1991; Moss et al. 2011). By promoting activity in ChIs, glutamate inputs could drive local striatal DA release via activating nAChRs on DA axons, as we observed here. This multi-synaptic event (Glu-ACh-DA) was insensitive to firing frequency of the glutamate inputs, which is in keeping with activation of ChIs as the intermediary: DA release is frequency insensitive when driven by direct activation of ChIs (Rice and Cragg 2004; Threlfell et al. 2010, 2012). We note that extrinsic sources of ACh input to the striatum from brainstem have recently been identified (Dautan et al. 2014) but, in pilot experiments we performed in collaboration, these inputs do not drive or otherwise modulate striatal DA release (not illustrated).

DA release driven here likely resulted from synchronization of activity in a small network of ChIs (Threlfell et al. 2012). ChIs are autonomous pacemakers (Wilson et al. 1990; Kawaguchi 1993; Bennett and Wilson 1998) but receive excitatory inputs from thalamus and cortex that can promote and even synchronize ChI firing (Consolo et al 1996b; Matsumoto et al. 2001; Reynolds and Wickens 2004; Lacey et al. 2007; Graybiel 2008; Ding et al. 2010; Schulz et al. 2011; Doig et al. 2014). We saw that DA release at some sites varied over time; and therefore, there is apparently moment-by-moment variation in the size of the ChI population recruited. This variability is consistent with the expectation that in cells such as ChIs, where spiking is predominantly driven by a strong autonomous pacemaker (Wilson et al. 1990; Kawaguchi 1993; Bennett and Wilson 1998), excitatory inputs would be expected to change spike probability by modifying the timing of spiking. We speculate that glutamate inputs might modulate DA signals in vivo variably according to the timing of their input relative to ChI activity phase, which will determine how large a population of ChIs can synchronously fire action potentials for driving DA release.

We show that M1/M2 cortical and Pf intralaminar thalamic inputs use different receptors for recruiting activity in ChIs and, in turn, for modulating DA release. Cortical inputs recruit predominantly AMPA receptors, while thalamic strongly recruit both AMPA plus NMDA receptors. The difference in balance of receptor types is consistent with the receptors shown to dominate the regulation of striatal ACh release and ChI activity after electrical stimulation (Consolo et al. 1997; Oswald et al. 2009), and with the relative dominance of receptors activated by these inputs to other striatal neurons, the spiny projection neurons, in some studies (Consolo et al. 1996a, 1996b; Smeal et al. 2008; Ellender et al. 2013) (but see Parker et al. 2016), although an ultrastructural explanation for these observations has yet to be resolved. This finding also supports the hypothesis that corticostriatal and thalamostriatal projection systems code information in temporally distinct ways (Ding et al. 2008; Doig et al. 2010). We saw that cortical inputs show a short latency and time window of operation, consistent with AMPA receptors having fast gating and desensitization. This brief window for summation of cortical inputs by ChIs will limit temporal summation (Götz et al. 1997), which could promote broadcasting of highly coincident cortical input to striatum (Agmon and Connors 1989; Helmchen et al. 1999). By contrast, thalamic inputs have a longer latency and more protracted time window for input summation and action potential generation, lasting tens of milliseconds, consistent with the slow gating of NMDA receptors. This large time window may lead to relatively long timescales over which inputs can be integrated (Götz et al. 1997; Gasparini et al. 2004) and might facilitate summation of inputs arriving within a relatively extended period. Information carried by individual Pf neurons, which may not arrive with perfect synchronization but in only relative temporal proximity due to characteristic low-frequency burst discharges of Pf neurons (Lacey et al. 2007), could nonetheless succeed in modulating ChI activity. Therefore, the different receptors and consequent input integration strategies used by ChIs could influence different components of action selection on different timescales.

In conclusion, cortical and thalamic inputs have effects on ChI excitability mediated by AMPA and NMDA receptors that can be sufficient to modulate striatal DA transmission via nAChRs. In turn, cortical and/or thalamic inputs might regulate behavior and plasticity partly via modulation of striatal DA, even in the absence of detectable changes in firing rate in DA neurons. There could be a greater range of functions for DA than predicted from the activities of DA neuron firing alone. In this regard, it is of note that a discrepancy has recently been identified between the DA signals detected in nucleus accumbens throughout an adaptive decision-making task and the underlying DA neuron firing rates expected from published literature (Hamid et al. 2015). These observations are consistent with the hypothesis that there are local striatal drivers of DA release, such as ChIs (Cachope et al. 2012; Threlfell et al. 2012), which we now show can be governed by corticostriatal and thalamostriatal inputs. Our findings might therefore have significant implications for the regulation of behavior and for dysfunction in disorders that extend beyond Parkinson's disease to those involving dysfunctional corticostriatal inputs including Huntington's disease, attention-deficit and hyperactivity disorder (ADHD), obsessive-compulsive disorder and addiction, whose management includes targeting DA.

## Funding

Medical Research Council UK (grant number MR/K013866/1); Parkinson's UK (grant numbers G1305, J-0901 Monument Trust Discovery Award); and a Clarendon Fund Studentship (to P.K.).
